# Impact of Organic Carbon Electron Donors on Microbial Community Development under Iron- and Sulfate-Reducing Conditions

**DOI:** 10.1371/journal.pone.0146689

**Published:** 2016-01-22

**Authors:** Man Jae Kwon, Edward J. O’Loughlin, Maxim I. Boyanov, Jennifer M. Brulc, Eric R. Johnston, Kenneth M. Kemner, Dionysios A. Antonopoulos

**Affiliations:** 1 Biosciences Division, Argonne National Laboratory, Argonne, IL, 60439, United States of America; 2 Korea Institute of Science and Technology (KIST) – Gangneung Institute, Gangneung, 210–340, S. Korea; 3 Institute for Genomics and Systems Biology, Argonne National Laboratory, Argonne, IL, 60439, United States of America; The University of Akron, UNITED STATES

## Abstract

Although iron- and sulfate-reducing bacteria in subsurface environments have crucial roles in biogeochemical cycling of C, Fe, and S, how specific electron donors impact the compositional structure and activity of native iron- and/or sulfate-reducing communities is largely unknown. To understand this better, we created bicarbonate-buffered batch systems in duplicate with three different electron donors (acetate, lactate, or glucose) paired with ferrihydrite and sulfate as the electron acceptors and inoculated them with subsurface sediment as the microbial inoculum. Sulfate and ferrihydrite reduction occurred simultaneously and were faster with lactate than with acetate. 16S rRNA-based sequence analysis of the communities over time revealed that *Desulfotomaculum* was the major driver for sulfate reduction coupled with propionate oxidation in lactate-amended incubations. The reduction of sulfate resulted in sulfide production and subsequent abiotic reduction of ferrihydrite. In contrast, glucose promoted faster reduction of ferrihydrite, but without reduction of sulfate. Interestingly, the glucose-amended incubations led to two different biogeochemical trajectories among replicate bottles that resulted in distinct coloration (white and brown). The two outcomes in geochemical evolution might be due to the stochastic evolution of the microbial communities or subtle differences in the initial composition of the fermenting microbial community and its development via the use of different glucose fermentation pathways available within the community. Synchrotron-based x-ray analysis indicated that siderite and amorphous Fe(II) were formed in the replicate bottles with glucose, while ferrous sulfide and vivianite were formed with lactate or acetate. These data sets reveal that use of different C utilization pathways projects significant changes in microbial community composition over time that uniquely impact both the geochemistry and mineralogy of subsurface environments.

## Introduction

Biogeochemical cycling of C is intimately coupled with the biogeochemical cycles of major (e.g., O, N, Fe, S), minor (e.g., Mn, Se), and redox active heavy metal and radioactive contaminant (e.g., Cr, As, Hg, U) elements through a complex network of electron donor/acceptor couples that drive much of the biogeochemical activity in surface and near-subsurface environments. For example, many chemolithoautotrophic microbes use reducing equivalents generated by the oxidation of Fe(II) or reduced sulfur compounds to fix CO_2_. Conversely, in marine sediments, dissimilatory sulfate reduction (DSR) and dissimilatory iron reduction (DIR) account for, on average, 62 ± 17% and 17 ± 15%, of organic C (OC) oxidation, respectively [1], and in freshwater sediments DIR can account for upwards of 70% of anaerobic C metabolism [2, 3]. Although much is known of the coupling of C, Fe, and S biogeochemistry in general, many fundamental aspects relevant to subsurface environments have yet to be elucidated, especially in the context of microbial community dynamics.

Dissimilatory iron-reducing bacteria (DIRB) are, in general, believed to outcompete dissimilatory sulfate-reducing bacteria (DSRB) for organic electron donors when microbially reducible Fe(III) (hydr)oxides are available [4, 5]. However, several studies reported that both DIR and DSR can occur concurrently in natural environments [6–9], and reactive transport model simulations by Bethke *et al*. (2008) indicate that that DIR and DSR can overlap when electron donor concentrations are in stoichiometric excess of available Fe(III). Moreover, Fe(II) resulting from DIR can react with sulfide from DSR to form FeS, and precipitation of FeS phases can promote further DIR and DSR by decreasing product inhibition. Further complicating the relative dynamics of DIR and DSR is a significant overlap in the utilization of specific organic electron donors between DIRB and DSRB. For example, various DIRB (e.g., *Geobacter* and *Anaeromyxobacter*) and DSRB (e.g., *Desulfobacter*) can utilize acetate, while *Shewanella* and *Anaeromyxobacter* (DIRB) and *Desulfovibrio* (DSRB) can utilize lactate as electron donors [10–12].

The reducing equivalents available to support DIR and DSR are ultimately derived from reduced OC present as a highly diverse pool of substrates ranging from easily assimilated low molecular mass acids and alcohols to complex biopolymers such as carbohydrates, proteins, and lipids that are generally not suitable electron donors for typical Fe(III)- and sulfate-reducing microorganisms until they are broken down to monosaccharides, amino acids, and short-chain aliphatic acids. The diversity of reduced OC forms available as electron donors for DIR and DSR provides the potential for a range of metabolic pathways for coupling the oxidation of C to the reduction of Fe(III) and sulfate and for the development of distinct microbial populations. Indeed, the availability of specific organic electron donors has been shown to affect development of different microbial populations under Fe(III)-reducing conditions [13–19]. However, few studies have focused on the effects of specific organic electron donors on the dynamics of Fe(III) and sulfate reduction [e.g., [14, 15, 17] from above], and fewer still provide intensive coincident monitoring of geochemical (C, Fe, and S) and microbial community dynamics [e.g., [17]].

The combination of the metabolic diversity of the microbial population and availability of specific organic electron donors determines the metabolic pathways by which DIR and DSR can proceed in a given system. However, the heterogeneity and complexity of geochemical conditions and microbial populations in environmental matrices makes predicting the dynamics of DIR and DSR difficult and requires coincident monitoring of microbial populations with relevant geochemical parameters pertaining to C, Fe, and S transformations. In this study, we examine the effects of organic electron donor availability (acetate, lactate, or glucose) on the dynamics of DIR and DSR and concomitant changes in microbial populations over time in microcosms containing both Fe(III) (as ferrihydrite) and sulfate that were inoculated with the native microbial populations present in subsurface sediment. We used a combination of next-generation DNA sequencing-enabled microbial community analysis and a suite of geochemical and mineralogical characterizations and found that the type of electron donor available produced distinct microbial community developmental trajectories and also influenced iron and sulfate reduction in these systems.

## Materials and Methods

### Experimental system

Kenneth Williams graciously provided the subsurface sediment used in this study which was obtained from the U.S. Department of Energy Office of Biological and Environmental Research’s Integrated Field Research Challenge (IFRC) site in Rifle, CO (39°32'19.0"N 107°43'26.1"W) [4, 20, 21]. The IFRC at Rifle, CO, is a multidisciplinary, multi-institutional project managed by Pacific Northwest National Laboratory (PNNL) and granted permission for sample acquisition. Briefly, the site is located on a small (~9 hectare) floodplain in northwestern Colorado, underlain by an aquifer comprising 6.5 to 8.5 m of unconsolidated sands, silts, clays, and gravels deposited by the adjacent Colorado River. Sediments were recovered from a depth of ~7 m below ground surface during installation of groundwater monitoring well LR-MLS-21 as described by Williams 2011 [21]. The well is located on the western portion of the floodplain, in an area minimally impacted by residual contamination from U- and V-bearing mill tailings (now removed). Groundwater at the site typically contains 6–10 mM sulfate [4, 21]. The native microbial community in the sediment (stored in the dark under anoxic conditions at 4°C) was used as the inoculum for the experiments described below.

The experimental system consisted of serum bottles of ~280 mL capacity. Subsurface sediment (10 g) from the Rifle IFRC, was added to the sterile bottles with a sterile spatula in an anoxic glove bag (4–6% H_2_ in N_2_). Next, 180 mL of sterile buffered medium equilibrated with 80:20 (vol/vol) N_2_:CO_2_ was added, and the bottles were sealed with Teflon-lined rubber septa and aluminum crimp caps. The basal anoxic medium consisted of (g L^-1^ unless specified otherwise) NaHCO_3_ (2.5), NH_4_Cl (0.25), NaH_2_PO_4_·H_2_O (0.6), KCl (0.1), 1mL of 1 mM Na_2_SeO_4_, and 10 ml L^-1^ each of modified Wolfe’s vitamin and mineral mixtures [22]. The experimental bottles were sparged again with N_2_:CO_2_ (80:20) to remove residual H_2_ in the headspace. Then, either acetate, lactate, or glucose (20 mM) was added as the sole electron donor, followed by the addition of 50 mM Fe(III) as ferrihydrite—prepared by titrating 0.5 M FeCl_3_ to pH 7.5 by the dropwise addition of 1.0 M KOH [23]—and 10 mM sulfate as electron acceptors. All amendments were prepared from sterile, anoxic stock solutions and transferred with sterile needles and syringes that had been flushed with anoxic gas (N_2_:CO_2_ = 80:20). The final volume of suspension in each bottle was 200 mL. The bottles were incubated in the dark at 30°C with continuous mixing on a roller drum. All experiments were performed in duplicate.

Subsamples were collected periodically for microbial community and geochemical analysis with a sterile syringe and needle (flushed with anoxic N_2_:CO_2_ = 80:20) in an anoxic glove bag. For measurement of total Fe(II), 0.25 mL of the suspension was mixed with 0.75 ml of 1 M HCl. Total Fe(II) was measured by acid extraction for at least 24 h followed by centrifugation at 25,000 × g for 10 min. Approximately 0.5 mL of the suspension was collected and centrifuged at 25,000 × g for 10 min for measurement of aqueous Fe(II), anions, and electron donor concentrations. For aqueous Fe(II), 0.2 mL of the supernatant was mixed with 0.2 mL of 1.0 M HCl, and 50 μL of supernatant was mixed with 0.95 mL of 15% isopropanol and stored under refrigeration for anion and electron donor analysis. Less than 0.5 mL of the suspension was collected for measurement of solution pH. Aliquots (2 mL) of the suspension collected for community analysis were dispensed into sterile microcentrifuge tubes that were then stored at –80°C until the experiments were completed. Aliquots (3 mL) of the suspensions were also collected at the end of the experiment and assayed for solid-phase Fe and S speciation by pXRD and X-ray absorption fine structure (XAFS) spectroscopy.

### Geochemical analysis

Ferrihydrite reduction was monitored by measuring the concentration of aqueous Fe(II) and total Fe(II) (total Fe(II) is comprised of aqueous Fe(II) and Fe(II) extracted from the solids by 0.75 M HCl) with the ferrozine assay [24]. Briefly, 1 mL of HEPES (50 mM)-buffered ferrozine reagent [25] was added to 0.05 mL of sample, and the Fe(II) concentration was measured at 562 nm with a spectrophotometer. The concentrations of sulfate and phosphate in samples were determined by using an ion chromatograph (Dionex 3000) with an IonPac AS11 analytical column (250 x 2 mm, Dionex) and 1–20 mM KOH at a flow rate of 0.5 mL min^-1^ (Dionex Corporation, Sunnyvale, CA, USA). The concentrations of glucose and acetate, lactate, and other organic acids were measured by high-performance liquid chromatography (HPLC) with an Agilent 1100 series HPLC equipped with refractive index and ultraviolet absorbance detectors (Aglient Technologies, Santa Clara, CA, USA). The samples were diluted with an equal volume of 10 mM H_2_SO_4_, and 50 μL of the diluted sample was injected on a Bio-Rad Aminex HPX-87H ion-exchange column (7.8 × 300 mm; Bio-Rad Laboratories, Hercules, CA, USA). The column was eluted isocratically with 5 mM H_2_SO_4_ at a flow rate of 0.6 mL min^-1^ at 50°C, with analyte detection at 210 nm. The aqueous-phase pH was measured by Semi Micro pH electrode (Thermo Scientific, Waltham, MA, USA).

### X-ray Absorption Fine Structure (XAFS) spectroscopy

The solid phases of the reactors were collected at day 124 and analyzed by XAFS spectroscopy to determine the chemical speciation of Fe. Three mL (for acetate and lactate systems) or 10 mL (for glucose system) of the suspensions were filtered through a 0.22 μm filter and the hydrated solids were sealed between layers of Kapton film in an anoxic glove box. XAFS measurements at the Fe K-edge (7,112 eV) were performed at the MRCAT/EnviroCAT 10-BM beamline [26] at the Advanced Photon Source, Argonne National Laboratory. Anoxic conditions were maintained during data collection by purging the sample chamber with N_2_. The energy of the incident X-rays was scanned using a Si(111) water-cooled double-crystal monochromator. Harmonic content was removed by detuning the second crystal to 50% of the maximum intensity. Data were collected in transmission mode using gas-filled ionization detectors. Monochromator energy calibration was maintained by the simultaneous collection of spectra from a metallic Fe standard using x-rays transmitted through the samples. The final spectrum for each sample was produced by averaging 3–5 consecutive scans. The extended XAFS (EXAFS) region of the spectrum was extracted using the program Autobk [27]. The contribution of spectroscopically-distinct Fe species in the k^3^-weighted EXAFS data was quantified using linear combination (LC) analysis implemented in the program ATHENA [28]. In addition to 2-line ferrihydrite, goethite, and lepidocrocite, spectra from the following reduced iron phases were considered as possible components in the LC analysis: vivianite, magnetite, Fe(II) adsorbed to carboxyl-functionalized beads at pH 7, siderite, green rust, and amorphous mackinawite. The standard of Fe(II) adsorbed to carboxyl-functionalized beads [29] was assumed to represent “disordered Fe(II) phases”, inclusive of Fe(II) adsorbed to cells or any disordered Fe(II) precipitates that do not have defined outer-shell structure in their EXAFS spectrum. The best-fit combination was chosen based on the quality of the fit as determined by the lowest reduced-chi-square value. Linear combination analyses were also performed on the derivative of the x-ray absorption near edge structure (XANES) data to corroborate the results of the EXAFS analysis.

### Synchrotron-based powder X-ray diffraction (pXRD)

The same samples that were analyzed by XANES and EXAFS were also analyzed by pXRD. Data were collected in a transmission geometry using a 17.5 keV incident photon beam (700 x 700 micron beam size) and a MAR165 CCD detector (165 mm diameter, 16-bit dynamic range, 80 x 80 micron pixel, detector located approximately 20 cm behind the sample). The two-dimensional patterns were integrated to Intensity vs. 2θ data using the fit2D software (http://www.esrf.eu/computing/scientific/FIT2D/). A pattern obtained from LaB_6_ was used for angular calibration of the experimental geometry. Data were analyzed by using the Jade 9.0 software (Materials Data, Inc.) and the standard patterns were obtained from the International Centre for Diffraction Data (ICDD) PDF-4^+^database.

### 16S rRNA-based analysis of microbial communities

Frozen suspensions stored at -80°C were thawed in a digital dry bath (Labnet International, Edison, NJ, USA) at 70°C for 10 min to maximize the efficiency of DNA extraction. Solid suspension (1 mL; approximately 0.05 g of solid) was used for extraction of total genomic DNA with a Power Soil DNA extraction kit (MO BIO, Carlsbad, CA, USA) and a bead-beating apparatus used according to the manufacturer’s directions. DNA concentration was quantified with a fluorometer (Qubit, Invitrogen, Carlsbad, CA, USA).

Amplicon libraries targeting the 16S rRNA encoding gene were sequenced using the 454 GS-FLX to obtain deep surveys of the microbial communities at an approximate depth of 6,000 sequences per sample, after quality filtering (S1 Table). PCR primers specific for the V3-V4 region of the 16S rRNA encoding gene containing 454-specific adapter sequences, as well as a 10-bp barcode, were used. The primers spanned *Escherichia coli* positions 338–802 using 338F (5’-ACTCCTACGGGAGGCAGC-3’) and equimolar amounts of the 802R reverse primers (802R-A 5’-TACCRGGGTHTCTAATCC-3’, 802R-B 5’-TACCAGAGTATCTAATTC-3’, 802R-C 5’-CTACDSRGGTMTCTAATC-3’, 802R-D 5’-TACNVGGGTATCTAATCC-3’) from the Ribosomal Database Project’s (RDP’s) pyrosequencing pipeline (http://pyro.cme.msu.edu/; [30]). Sequencing was performed by the Next Generation Sequencing (NGS) Core at Argonne National Laboratory using the GS-FLX LR70 sequencing chemistry (454 Roche Applied Science, Branford, CT, USA). The data are publicly available via a project specific page in MG-RAST based at Argonne, including instant availability of the sequence data, bioinformatic analyses and tools (http://metagenomics.anl.gov/linkin.cgi?project=8373; MG-RAST IDs 4556032.3–4556077.3).

Sequence data were processed and analyzed using QIIME, version 1.5.0 [31]. Operational taxonomic units (OTUs) were picked at 97% sequence identity with uclust [32]. Representative sequences were chosen from each OTU by selecting the most abundant sequence in that OTU, aligned using PyNAST [33], and then assigning taxonomy with the Ribosomal Database Project’s Classifier tool. The PyNAST-aligned sequences were also used to build a phylogenetic tree with FastTree [34], and diversity metrics for beta-diversity (including UniFrac distances; [35]) were then calculated between all samples for ordinations.

## Results and Discussion

### Overall changes in microbial community structure and geochemical variation over time according to electron donor

**Fig 1:**
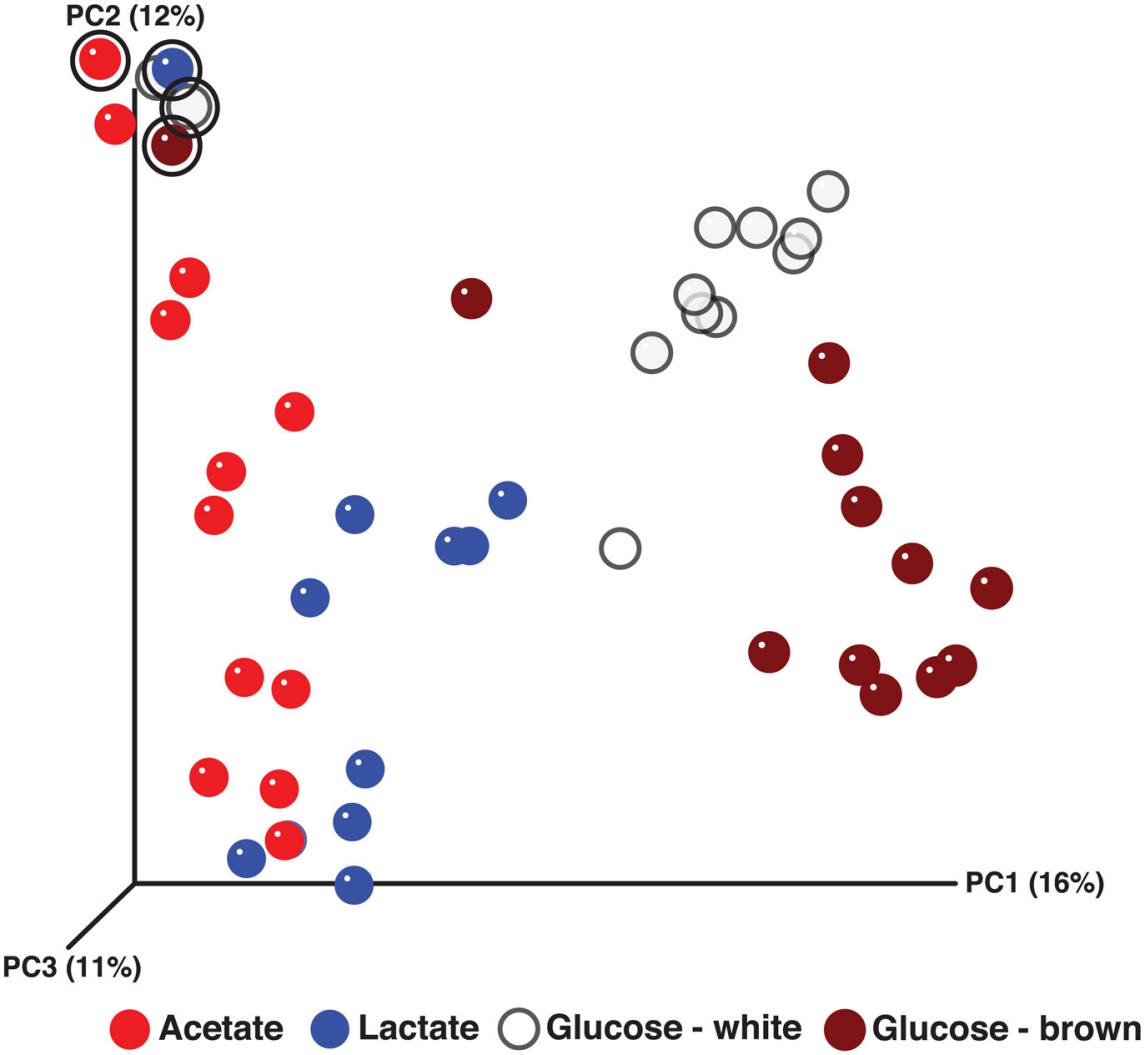
Principal coordinates analysis (PCoA) of bacterial communities based on a 454 16S rRNA-based amplicon library data (unweighted UniFrac distance). The initial sample points (circled in bold black) from all four systems are localized in the upper left quadrant of the ordination. From there, subsequent sample points for all incubations are distributed along the first principal coordinates axis (PC1), to varying degrees, on the basis of the specific electron donor. The PCoA indicates that the bacterial community structure from each incubation can be classified into one of four groups according to the availability of the specific electron donor (acetate, lactate, or glucose [“white” or “brown”]), with the glucose groups being farthest from the initial composition.

Amplicon library inventories targeting the 16S rRNA encoding gene and sequenced using 454-based technology were generated from each of the electron donor-amended incubations over time. QIIME-enabled analysis of the amplicon library sequencing data showed that the compositions of the microbial community and abundances at day 0 were relatively similar between incubations (S2 Table). The major phyla initially within each electron donor-amended incubation were Actinobacteria (17–31% of the total community membership across the incubations), Bacteriodetes (27–43%), Firmicutes (7–10%), and Proteobacteria (29–30%). Members of these phyla that were consistently found across all of the incubations at day 0 and could be assigned confidently at the genus level included *Arthrobacter* (2–10%; Actinobacteria), *Leifsonia* (11–19%; Actinobacteria), *Flavobacterium* (22–41%; Bacteroidetes), *Desulfosporosinus* (1–2%; Firmicutes), *Pseudomonas* (10–13%; Proteobacteria), and *Rhodoferax* (3–6%; Proteobacteria). Analysis of the amplicon library inventories provided a global perspective of the gross changes in microbial communities between electron donor-amended incubations. Principal coordinates analysis (PCoA) of unweighted UniFrac distances between the individual sample points indicated that the bacterial communities in each incubation changed in composition according to the availability of specific electron donors over time (Fig 1). Community composition in the acetate-amended incubation did not exhibit as great of a shift from the initial sample point (without donor) as the other incubations. The lactate-amended community appeared to shift away from the acetate-amended community, whereas the samples that were most distant from the initial sample point were from the glucose-amended incubations. Interestingly, replicate incubations with glucose showed diverging microbial community development trajectories, as well as different suspension colors—subsequently referred to as glucose “brown” (GB)or glucose “white” (GW) based on the color of the suspensions (S1 Fig).

**Fig 2:**
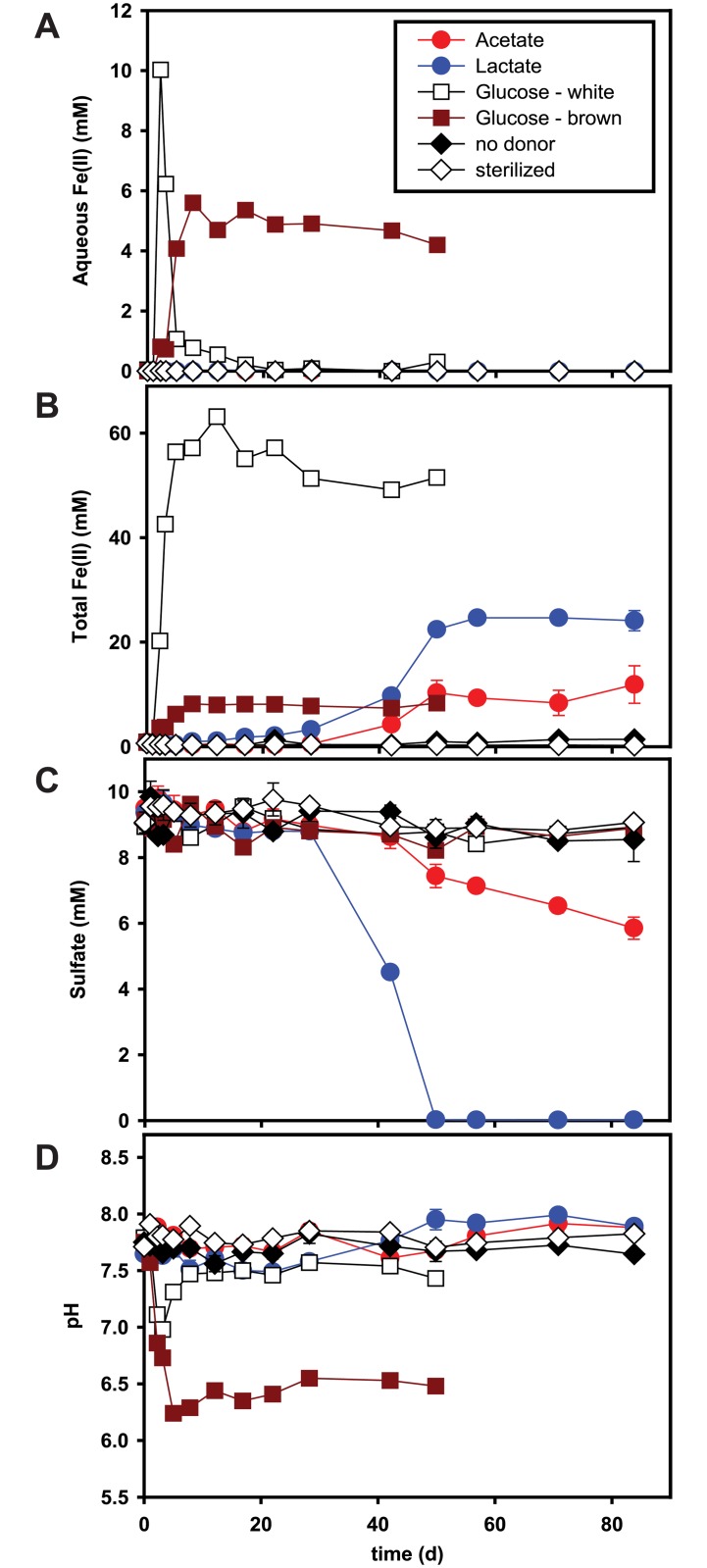
Variation of aqueous Fe(II) concentration (panel a), total Fe(II) (panel b), sulfate concentration (panel c), and pH (panel d) over time. The incubations were started with 20 mM of electron donor (acetate [red circle], lactate [blue circle], glucose “white” [white square], glucose “brown” [brown square]), 50 mM of two-line ferrihydrite, and 10 mM of sulfate. Results are the means of duplicate analyses, and bars indicate one standard deviation (except for glucose “white” and “brown” data, which are based on single analyses). No donor control denoted by black diamond, sterilized control by white diamond.

Aqueous Fe(II), total Fe(II), sulfate, and pH were monitored in each incubation over time (Fig 2). Aqueous Fe(II) did not accumulate in acetate- or lactate-amended incubations (Fig 2A). However, total Fe(II) increased slowly and accumulated up to 11 mM and 25 mM in acetate- and lactate-amended incubations, respectively (Fig 2B). Sulfate reduction occurred concurrently with ferrihydrite reduction in both acetate- and lactate-amended incubations (Fig 2C). In lactate-amended incubations, sulfate reduction (ca. 10 mM) was completed within 50 d, while only 4 mM of sulfate was reduced with acetate at the end of the experiment (day 84). In the presence of glucose, ferrihydrite reduction was initiated within 2 d. Each of the two replicates with glucose exhibited different colors (white versus brown), as well as different Fe(II) production profiles. Because of this inconsistency in duplicate incubations, additional incubations were created in triplicate under the same conditions. Again, a divergence in biogeochemical outcome was observed, where two of the three exhibited lighter precipitates and one had darker precipitates (S2 Fig). The GW incubation showed rapid increases in aqueous Fe(II) and total Fe(II) (Fig 2A and 2B). However, aqueous Fe(II) fell below the detection limit after 20 d, and total Fe(II) remained stable at around 50 mM. In the GB incubation aqueous Fe(II) and total Fe(II) accumulated up to 5 mM and 8 mM, respectively, and remained stable for 50 d. In the repeat of the glucose incubations, the replicates with the lighter precipitates (designated as Glucose 1 and Glucose 3 in S2 Fig) exhibited total Fe(II) profiles similar to the GW incubation and the replicate with the darker precipitates (Glucose 2 in S2 Fig) was similar to the GB incubation. Total Fe(II) accumulated faster with glucose than with acetate or lactate (Fig 2B).

The pH in the acetate-amended incubation was relatively stable between 7.6 and 7.9 throughout the experiments, while the pH in the lactate-amended incubations decreased slowly to 7.5 for 28 d, then increased to 8 within 50 d (Fig 2D). Initially, the pH in both GW and GB incubations rapidly decreased from 7.8 to below 7. However, the pH in GW incubation increased after 3 d and then remained stable at 7.5, while the pH in the GB incubation stabilized at around 6.5.

### Mineral transformation

**Fig 3:**
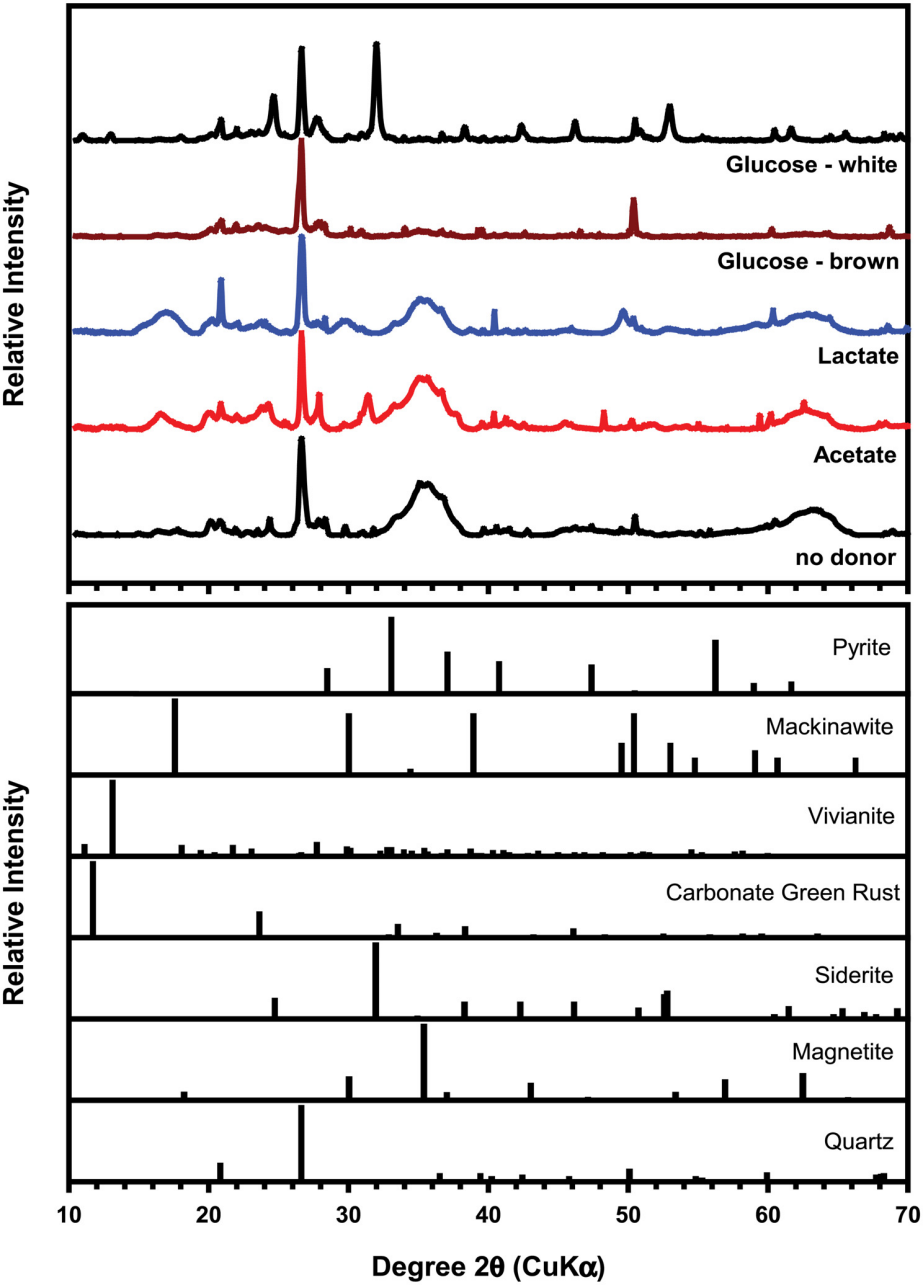
Synchrotron-based X-ray diffraction (XRD) patterns of the solids in the incubations at 124 d after incubation (top) and reference patterns of select minerals (bottom).

The synchrotron-based powder X-ray diffraction (pXRD) patterns of the solids in all incubations at day 124 showed peaks from quartz due to the sandy sediment used as the inoculum (Fig 3). The two broad peaks at 36° and 64° 2θ are consistent with to two-line ferrihydrite [23], indicating that a significant amount of the parent Fe(III) oxide remained after incubation with acetate or lactate, but not with glucose. The GW incubation showed multiple peaks corresponding to siderite (FeCO_3_). The lack of other significant peaks in the patterns might be caused by the poor crystallinity or the nanoparticulate form of the secondary mineralization products. Fe K-edge x-ray absorption analysis (XANES and EXAFS) was employed to identify such minerals and quantify their contents (Table 1 and S3 Fig; [36, 37]).

**Table 1:** Mineral phases identified in incubations with ferrihydrite^a^.

		Mineral phase (as % of total Fe in the solids)^a^					
e- donor supplied	LC fit	Ferrihydrite^b^	Siderite	Vivianite	Disordered Fe(II)^c^	Green rust	FeS
Acetate	EXAFS	76	-	13	-	10	4
	XANES	75	-	11	-	11	4
Lactate	EXAFS	76	-	6	-	-	17
	XANES	74	-	8	-	-	15
Glucose (white)	EXAFS	13	64^d^	-	32^d^	-	-
	XANES	6	66^d^	-	27^d^	-	-
Glucose (brown)	EXAFS	84	-	2	4	-	-
	XANES	90	-	8	8	-	-

^a^ Based on linear combination (LC) fits of the XANES and EXAFS data with up to four of the listed standards. Components were not constrained to add up to 100%. Uncertainties reported by the numerical fitting procedure are <2%. Overall uncertainties are estimated at ±5%. Higher sensitivity to the structural differences between Fe(II) phases was observed in fits of the EXAFS data than in fits of the XANES data.

^b^ The spectrum used in the LC fit was obtained from the same 2-line ferrihydrite phase that was used in the reactors.

^c^ This component represents Fe(II) species that do not have a defined structure beyond the first O shell, e.g. disordered precipitates or Fe(II) adsorbed to the mineral or biological surfaces in the system. A spectrum from Fe(II) adsorbed to carboxyl-functionalized beads was used (details in SI section).

^d^ Crystalline siderite and adsorbed Fe(II) species were used as spectral endmembers to reproduce the data and to quantify the corresponding spectral content. We note that spectra from a fresh amorphous precipitate of Fe(II) in bicarbonate solution could also be fit with a linear combination of crystalline siderite and adsorbed Fe(II) species (not shown). While the data and fits demonstrate the presence of siderite, the noted fraction numbers may not represent the actual content of two distinct species in the sample, but possibly the predominance of an amorphous ferrous carbonate precipitate or a combination of the three species mentioned above. It is not possible to distinguish between these scenarios with a linear combination fit.

In lactate- or acetate-amended incubations, XANES and EXAFS analysis indicated the presence of a minor FeS phase (S3 Fig and Table 1). Formation of ferrous sulfide can be expected because of the concurrent iron and sulfate reduction observed in these systems (Fig 2). Production of sulfide by DSRB and the subsequent reductive dissolution of ferrihydrite, as well as precipitation of ferrous sulfide, might be the cause of this insoluble ferrous sulfide mineral. EXAFS analysis also suggested the formation of vivianite [Fe_3_(PO_4_)_2_], which can be expected in the presence of phosphate [38, 39].

**Fig 4:**
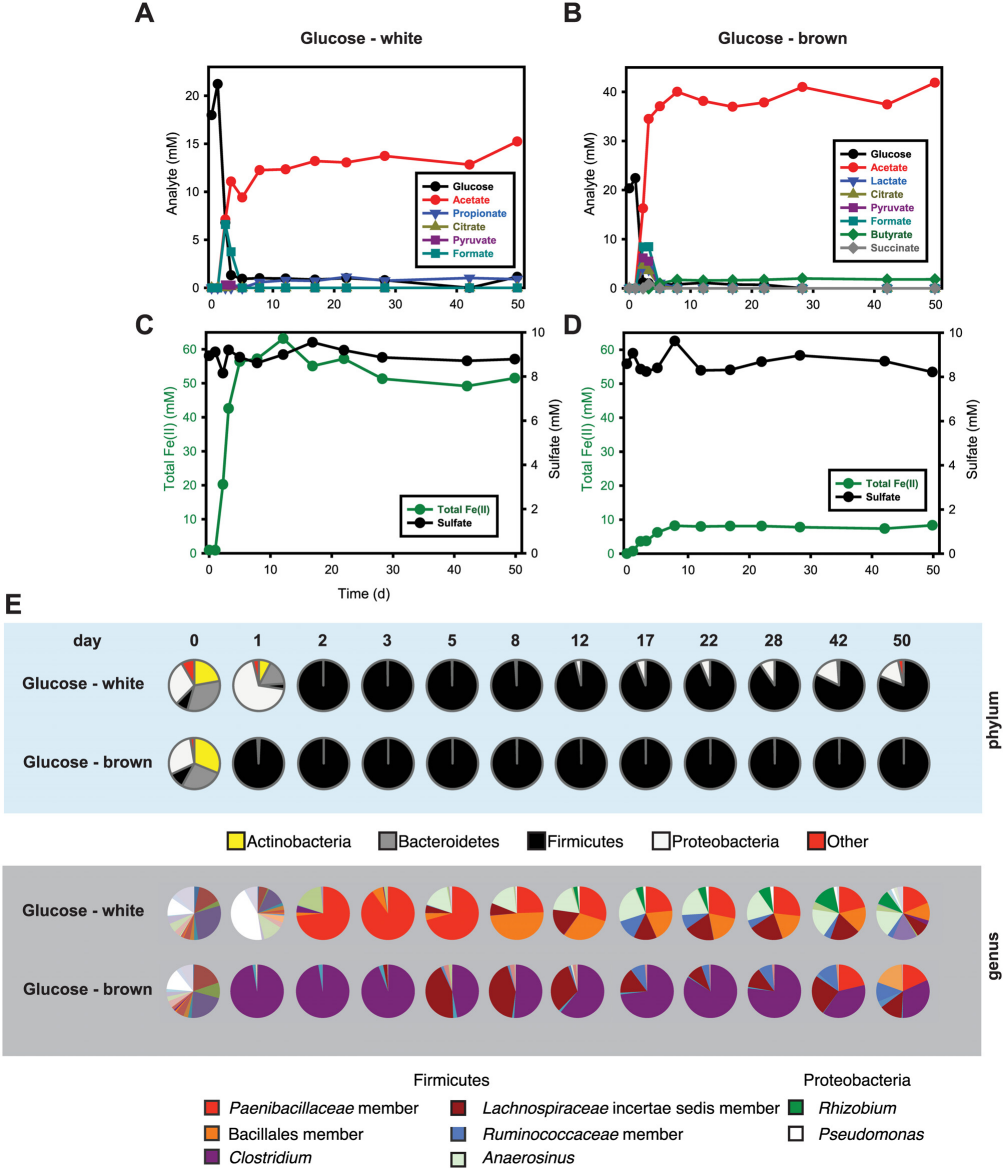
Glucose consumption and organic acid production (panel a) and total Fe(II) production and sulfate reduction (panel c) in the presence of glucose (20 mM), for the glucose “white” system (see main text). Glucose consumption and organic acid production (panel **b**) and total Fe(II) production and sulfate reduction (panel **d**) in the presence of glucose (20 mM), for the glucose “brown” system (see main text). Both incubations were started with 50 mM of two-line ferrihydrite and 10 mM of sulfate. The data are based on a single replicate for each bottle. Bacterial community changes over time are shown as pie charts in panel **e**. Again, similar compositions at day 0 for both communities (leftmost pie chart) contrast with the distinct community compositions observed for the two systems at both the phylum and genus levels. Labeled organisms are those discussed further in the main text.

In glucose-amended incubations, ferrihydrite reduction was concurrent with the fermentation of glucose. Fe(II) sulfide minerals were not detected by pXRD or XAFS in either the GW or GB incubations (Fig 3, S3 Fig, and Table 1), consistent with the lack of sulfate reduction in these systems (Fig 2C). Reduction of ferrihydrite in GW was complete and resulted in formation of siderite (FeCO_3_) and amorphous or adsorbed Fe(II) phases, whereas less than 20% of the ferrihydrite in GB was reduced, and only small amounts of vivianite and disordered Fe(II) were identified (Table 1). Siderite formation in GW is likely due to the production of high amounts of CO_2_ during glucose fermentation. An increase in pH back to 7.5 within 8 d suggested that CO_2_ was rapidly consumed and precipitated as siderite (Fig 2D). In GB, however, siderite did not form, likely because of lower CO_2_ production. A lower pH persisted because higher amounts of organic acids (i.e., acetate) were produced in GB than in GW (Fig 4).

### Acetate- and lactate-amended incubations

**Fig 5:**
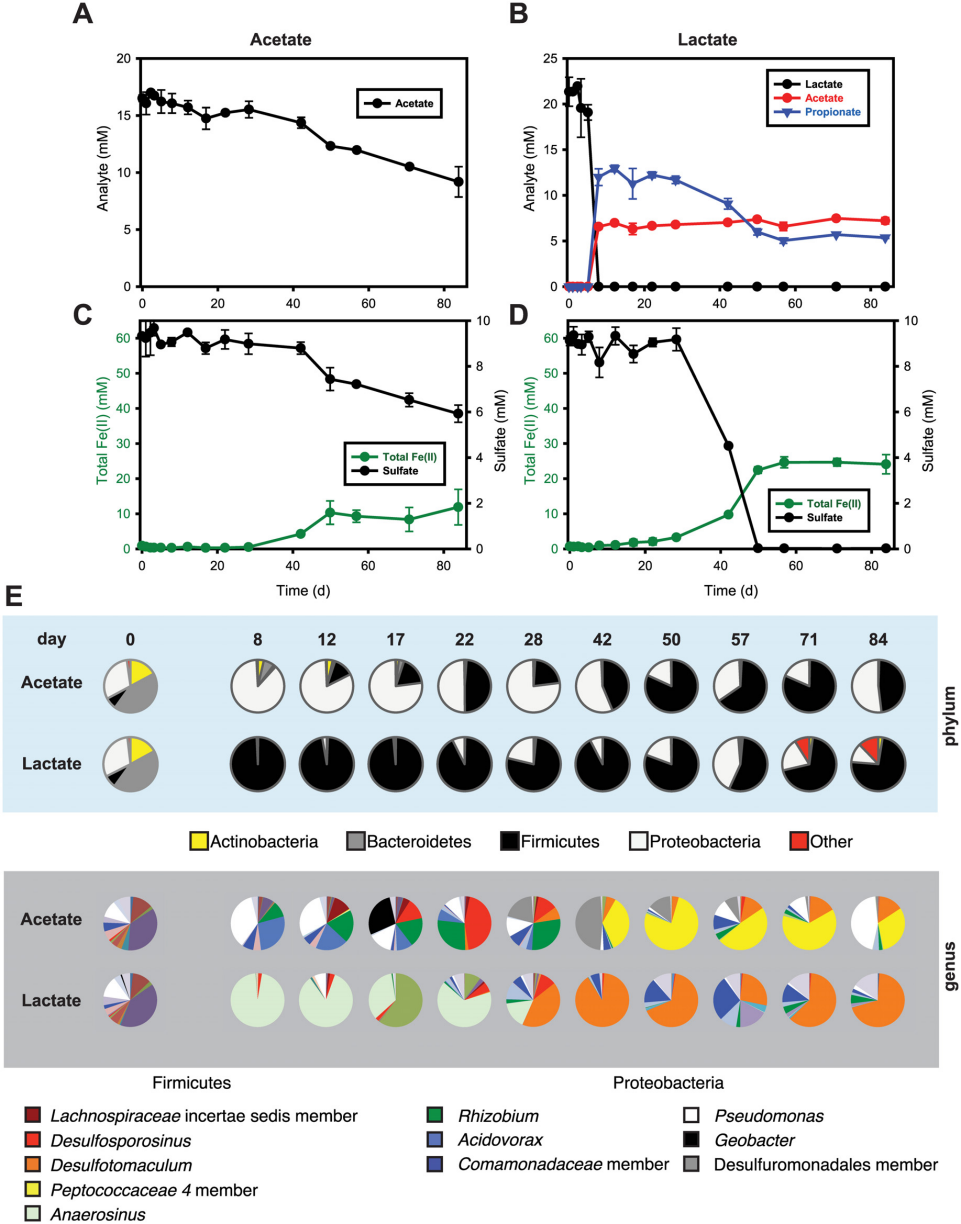
Acetate consumption (a) and total Fe(II) production and sulfate reduction (panel c) in the presence of acetate (17 mM), for incubations started with 50 mM of two-line ferrihydrite and 10 mM of sulfate. Lactate consumption and acetate/propionate production (panel **b**) and total Fe(II) production and sulfate reduction (panel **d**) in the presence of lactate (22 mM), for incubations started with 50 mM of two-line ferrihydrite and 10 mM of sulfate. Results are the means of duplicate analyses, and bars indicate one standard deviation. Bacterial community changes over time are shown as pie charts in panel **e**. Similar compositions at day 0 for both communities (leftmost pie chart) contrast with the distinct community compositions observed for the two systems at both the phylum and genus levels. Labeled organisms are those discussed further in the main text.

In the acetate-amended incubations, acetate was stable at around 15–17 mM for the first 28 d and then slowly decreased to 10 mM by the end of the experiment (Fig 5A). In terms of gross community composition, the Firmicutes decreased from 7.2% at day 0 to 1% at day 3 and then steadily increased to around 80% by the end of the experiment. In contrast, the Proteobacteria increased from 30.6% at day 0 to 79.9% by day 3 and remained highly abundant (17.8–87.4%) for the rest of the experiments.

In acetate-amended incubations, no evidence of Fe(III) and sulfate reduction was observed until day 28. Although a lag period of 28 d for the onset of Fe(III)/sulfate reduction is longer than expected based on prior studies involving acetate-amended sediments from the Rifle IFRC [4, 21, 40], longer lag periods are not uncommon [40, 41]. *Geobacter* did not appear to play a significant role in ferrihydrite reduction (exhibiting an overall low abundance initially, except at day 21). This was unexpected, given that *Geobacter* spp. are often highly abundant during both in situ and ex situ biostimulation of Rifle IFRC sediments by the addition of acetate [21, 42–44]. Instead, after 28 d, members of the family *Peptococcaceae* (a family within the Firmicutes), including *Desulfotomaculum* spp., were dominant, with acetate oxidation coupled with Fe(III) and sulfate reduction (Fig 5A, 5C and 5E). Several species in the genus *Desulfotomaculum* are known to reduce sulfate, coupled with oxidation of acetate to CO_2_ via an acetyl-CoA pathway [45–49]. The observed consumption of ~5 mM acetate and ~4 mM sulfate was in good agreement with the stoichiometry for sulfate reduction coupled with acetate oxidation (CH_3_COO^−^ + SO_4_^2−^ → 2HCO_3_^−^ + HS^−^), sulfide and bicarbonate being likely products, but not directly observed. Additionally, an increase (up to 47.8%) in a population in the order Desulfuromonadales (class Deltaproteobacteria) was observed between days 28 and 50, though the ability to reduce sulfate is not recognized among species in this order. However, a recent study observed that *Desulfuromonas* (a member of this order that is a recognized dissimilatory sulfur reducer but it is not known to reduce sulfate [50, 51]) was enriched in sediment incubations with sulfate versus those without [52]. It is possible that the formation of S(0) as a product of Fe(III) reduction by sulfide resulting from DSR stimulated this population [53–55].

Lactate stimulated primarily members of the Firmicutes and (to a lesser degree) the Proteobacteria (Fig 5E; S2 Table). Lactate rapidly decreased from 20 mM to < 50 μM within 8 d, with simultaneous production of 7 mM of acetate and 13 mM of propionate over this same period (Fig 5B). Firmicutes increased from 7.6% at day 0 to 99.2% by day 8 and remained relatively stable in abundance for the duration of the experiments (55.4–98.4%), while Proteobacteria rapidly decreased from 30.6% at day 0 to 0.7% by day 8 and then slowly increased to 41.9% by day 22. Lactate can be fermented or oxidized (or both) by a variety of known subsurface microorganisms, such as *Propionibacterium* (a member of the Actinobacteria) or *Clostridium propionicum* (Firmicutes) [50, 56–58]. Our results showed a spike in the relative abundance of *Anaerosinus* (a member of the family *Veillonellaceae* within the Firmicutes). Although no identified species of *Anaerosinus* are known to ferment lactate, this capability is widely distributed among many other genera within the *Veillonellaceae*.

Propionate produced from lactate fermentation was subsequently oxidized with concurrent sulfate reduction (Fig 5B). Sulfate reduction concomitant with Fe(II) production was observed from day 28 through day 45, coupled to the oxidation of propionate and a spike in the relative abundance of a population of *Desulfotomaculum* (Fig 5B, 5D and 5E). Several *Desulfotomaculum* spp. capable of propionate oxidation coupled with sulfate reduction have been reported [59, 60]. The oxidation of ~6 mM propionate was observed concurrently with reduction of 9 mM sulfate; this is reasonably close to the stoichiometry of sulfate reduction coupled to complete propionate oxidation (4CH_3_CH_2_COO^−^ + 7SO_4_^2−^ → 12HCO_3_^−^ + 7HS^−^ + H^+^).

#### Coupling of dissimilatory sulfate and iron reduction in acetate- or lactate-amended incubations

The simultaneous reduction of sulfate and ferrihydrite and increases in sulfate-reducing bacteria (e.g., *Desulfotomaculum*) after 28 d in both the acetate-and lactate-amended incubations suggests that sulfate reduction itself might affect ferrihydrite reduction. In addition, both the abundance of *Desulfotomaculum* and the extent of sulfate reduction were higher in lactate-amended incubations than in acetate-amended incubations; an increase in the proportion of *Desulfotomaculum* with lactate (26.6–89.8%) versus the proportion observed with acetate (3.9–15.9%) is proportionate with the extent of sulfate reduction in each. EXAFS analysis in the current experiments also indicated the formation of 4% and 17% FeS (as mackinawite) in acetate- and lactate-amended incubations, respectively (Table 1 and S3 Fig). Several studies have reported that sulfide can reduce iron (hydr)oxides [61, 62] and the concomitant reduction of sulfate and production of Fe(II) along with observation of DSRB and ferrous sulfide minerals as the major secondary minerals suggests that ferrihydrite was reduced by sulfide produced by DSR (reactions Eqs 1–3):

$${\text{HS}}^{-}+\text{Fe}{\left(\text{OH}\right)}_{3}\left(\text{s}\right)+2{\text{H}}^{+}\to {\text{Fe}}^{2+}+{\text{S}}^{0}+3{\text{H}}_{2}\text{O}$$ (1)

$${\text{Fe}}^{2+}+{\text{HS}}^{-}\to \text{FeS}\left(\text{s}\right)+{\text{H}}^{+}$$ (2)

$$2{\text{HS}}^{-}+\text{Fe}{\left(\text{OH}\right)}_{3}\left(\text{s}\right)+{\text{H}}^{+}\to \text{FeS}\left(\text{s}\right)+{\text{S}}^{0}+3{\text{H}}_{2}\text{O}$$ (3)

Previous studies indicate that DIRB can outcompete DSRB for organic substrates and H_2_ when microbially reducible iron (hydr)oxides are available [63]. A more recent study showed that DIR and DSR can occur successively [64]. This study also found no synergetic effect between DIR and DSR; in fact, the simultaneous action of DIR and DSR decreased the rate and extent of iron reduction. However, several studies have reported that both DIR and DSR can occur concurrently in natural environments [6–9].

The reactive transport model simulation by Bethke *et al*., (2008) [6] showed that DIR and DSR overlap in the presence of a limited amount of iron (hydr)oxides and an excess of electron donor. The Fe(II) from DIR then reacts with sulfide from DSR to form an FeS precipitate, and therefore both DIR and DSR can proceed without product inhibition. Similarly, our experimental system included an excess of electron donor (enough to completely reduce both 50 mM Fe(III) to Fe(II) and 10 mM sulfate to sulfide) and showed simultaneous ferrihydrite and sulfate reduction, along with precipitation of ferrous sulfide in the presence of acetate or lactate. In a parallel study we observed that ferrihydrite reduction was limited and slow in the presence of low sulfate (0.2 mM) [65]. These results are consistent with Fe(II) production resulting from abiotic reduction of ferrihydrite by sulfide produced by reduction of sulfate by DSRB populations.

Three possibilities could explain the limited initial Fe(III) reduction in the presence of excess acetate and lactate: (1) low abundance of DIRB in the sediment used to inoculate the microcosms, because the relative proportion of the initial microbial community phylogenetically related to known DIRB (e.g., *Geobacter* spp. and *Pseudomonas* spp.) was less than 0.1% (Fig 5E); (2) outcompetition of DIRB by non-DIRB and/or enrichment of spore-forming organisms; or (3) inhibition of Fe(III) reduction by phosphate (~ 4 mM) in the medium, which interacts strongly with Fe oxides. Of these, inhibition of Fe(III) reduction by phosphate seems most likely as phosphate concentrations were much lower (8–250 μM) in studies showing rapid reduction of Fe(III) in Rifle IFRC sediments [4, 9, 17, 21, 66]. Sorption of phosphate in our microcosms was rapid with dissolved phosphate decreasing from 4 mM to 600 μM within minutes of assembling all of the system components and decreasing below 50 μM in all microcosms within 1 d. This indicates that most of the phosphate present was associated with solid phases, ferrihydrite in particular. Sorbed phosphate is known to alter surface reactivity or block access of bacterial cells to sites on the Fe(III) (hydr)oxide surface, thereby limiting the rate of bioreduction [67, 68]. In addition to phosphate, the sorption of other oxyanions such as silicate as well as dissolved organic carbon (e.g., humic substances and microbially produced extracellular polymeric materials) can significantly alter the rates of microbial Fe(III) oxide reduction as well as the nature of the Fe(II)-bearing secondary minerals that form [68–71] and may impact Fe(III) reduction in situ.

#### Two-stage ferrihydrite reduction in lactate-amended incubations

We observed a two-stage ferrihydrite reduction process in the lactate-amended incubations. Depletion of lactate within the first 5 d was concurrent with a slow increase in Fe(II) (0.14 mM d^-1^) without sulfate reduction (Fig 5B and 5D) and was followed by oxidation of propionate after 28 d that corresponded with rapid increases in both sulfate reduction and total Fe(II) (0.86 mM d^-1^). The initial slow rate of Fe(III) reduction might have been due to the use of Fe(III) as a sink for excess electrons [72, 73] during lactate fermentation by *Anaerosinus*, which by day 8 comprised 96.2% of the community. In the previous section, we described how *Desulfotomaculum* couples sulfate reduction with complete oxidation of propionate; *Desulfotomaculum* appeared after 28 d and became a dominant member of the community (up to 89.8% at day 42; Fig 5E). These results suggest that the initial iron-reduction-dominated phase might result from the growth of *Anaerosinus*, followed by a second sulfate/iron reduction phase likely due to *Desulfotomaculum*.

The population of *Desulfosporosinus* in both the acetate- and lactate-amended incubations appeared at day 17, peaked at day 22, and disappeared by day 42. The appearance of *Desulfosporosinus* during this period is curious given that cultivated representatives of this genus have not been reported to use acetate as an electron donor for anaerobic respiration, and acetate concentrations in both acetate- and lactate-amended incubations were stable (i.e., no indication of acetate utilization). However, other studies have reported increases in the abundance of *Desulfosporosinus* spp. following acetate amendment of subsurface sediments [17, 74, 75]

### Glucose-amended incubations

The glucose-amended incubations showed different kinds and amounts of organic acid production (Fig 4A and 4B). The GW incubation showed transient accumulation of citrate (0.1 mM), pyruvate (0.3 mM), and formate (6.6 mM) within 5 d and accumulation of propionate and acetate up to 1 mM and 15 mM for 50 d, respectively. In GB, lactate (4.0 mM), citrate (4.7 mM), pyruvate (6.2 mM), formate (8.5 mM), and succinate (0.8 mM) transiently accumulated within 5 d, and accumulation of 2 mM butyrate and 40 mM acetate was observed at the end of the experiment. Variability in glucose fermentation product distributions were also observed among the triplicate incubations of the repeat of the glucose-amended microcosms (S2 Fig). Two distinctly different microbial communities developed in GW and GB. The abundance of Firmicutes in GB increased from 10.0% to 98.5% within 1 d and remained at almost 100% throughout the experiments, to the exclusion of other phyla (Fig 4E; S2 Table). In GW, Firmicutes increased from 7.9 to 99.9% within 2 d and slowly decreased to 80.9% at day 50, while Proteobacteria in GW increased from 29.3% at day 0 to 68.8% at day 1 before decreasing to 0.03% at day 2. The proportion of Proteobacteria then slowly increased to 15.5% by day 50.

Faster accumulation of total Fe(II) with glucose versus acetate or lactate suggested that glucose-fermenting bacteria grew faster than acetate- or lactate-utilizing bacterial communities. However, replicate glucose bottles followed different biogeochemical trajectories (Figs 1 and 4, and S2 Fig). All systems exhibited rapid fermentation of glucose in the first 10 d, leading to different distributions of mixed-acid fermentation products (Fig 4A and 4B, and S2 Fig), consistent with known variations in glucose fermentation pathways. The communities shifted rapidly and remained relatively constant for the rest of the experiment (Fig 4); however, the community profiles in the incubations with glucose were very different between replicates (Fig 4E and S2 Table). The GW system exhibited a maximum accumulation of Fe(II) by day 12 and was dominated at day 2 by a population in the family *Paenibacillaceae*, while the GB system had a maximum accumulation of Fe(II) by day 8 and was dominated by populations identified as *Clostridium* (day 1) and *Lachnospiraceae* (unclassified) (day 5).

Initially at day 0, *Clostridium* and *Lachnospiraceae* (unclassified) were present at about 0.9% and 0.1%, respectively, in GB, but were nearly undetectable (< 0.0%) in GW. In contrast, *Anaerosinus* was present at about 0.4% in GW but was nearly undetectable (< 0.0%) in GB. Glucose fermentation via these microorganisms can occur through many different metabolic pathways in a mixed microbial community (e.g., sediment from a natural environment). For example, fermentative microorganisms metabolize glucose to acids, alcohols, and hydrogen, which are in turn oxidized by a variety of indigenous microorganisms such as DIRB and DSRB [76]. In fact, our glucose-amended incubations showed various fermentation products (Fig 4A and 4B). Most notably, the production of acetate per mol of glucose consumed was much higher in GW than in GB. Carbon recovered in fermentation end products (organic acids only) at day 50 represented 25% and 73% of the C added as glucose in GW and GB, respectively, suggesting that other fermentation products such as CO_2_ and alcohols were also produced. In fact, the rapid increase in headspace pressure of the bottles with glucose, especially GW, indicated the release of CO_2_ and/or H_2_ over time. Also, a comparison of the variation of aqueous Fe(II) with pH between GW and GB suggests that accumulation of aqueous Fe(II) was due to the low pH in glucose-amended incubations (Fig 2). Glucose fermentation can reduce pH via CO_2_ and organic acid production. The decrease in aqueous Fe(II) in GW corresponded to the increase in pH. Likewise, little to no accumulation of aqueous Fe(II) in the acetate- or lactate-amended incubations suggested that aqueous Fe(II) was precipitated at pH > 7.5 (Fig 2A and 2D).

Concentrations of acetate in GW and GB accumulated up to 13 and 40 mM, respectively, after 5 d, while total Fe(II) accumulated up to 50 and 8 mM (Fig 4A and 4B). Given that *Clostridium* in GB rapidly increased to 96.6% at day 1, this genus was likely a major driver for the lower accumulation of total Fe(II). Others have reported that Fe(III) reduction via fermentative microorganisms such as *Clostridium* spp. is not an energy-conserving process and that less than 5% of the reducing equivalents are transmitted to Fe(III) [77, 78]. Conversely, members of the family *Paenibacillaceae* in GW increased to 73.4% and 90.1% at day 2 and day 3, respectively. *Paenibacillus* and *Brevibacillus* spp. not previously shown to reduce Fe(III) were found to be predominant in acidic subsurface sediments contaminated with U(VI)[19]. In addition, cell surface ferrireductase activity and extracelluar reductant secretion by *Paenibacillus polymyxa* have been reported [79]. A recent study also demonstrated that a *Paenibacillus* sp. isolated from subsurface sediment biofilms reduced soluble Fe(III) complexes at a rate similar to that for *Shewanella alga* BrY but did not reduce poorly crystalline ferrihydrite [80]. Although *Paenibacillus* sp. did not reduce solid-phase iron hydroxides without an electron transfer mediator (e.g., anthraquinone-2,6-disulfonate [AQDS]), other species or strains in this class might be able to reduce ferrihydrite significantly.

### Summary

The results of this study demonstrate that specific electron donors can strongly affect the rates and extent of Fe(III) and sulfate reduction, changes in microbial community compositions, and the resulting mineralogy of Fe-bearing minerals. Reduction of Fe(III) (hydr)oxides and sulfate by DIRB and DSRB can yield a suite of Fe(II) and S(-II) species including soluble Fe(II) complexes, Fe(II) complexes with the surfaces of organic and inorganic solid phases, a host of mineral phases containing structural Fe(II) (e.g., magnetite, siderite, green rust), and insoluble ferrous sulfide, by the reaction of sulfide by DSRB with Fe^2+^ resulting from DIR (e.g., makinawite, greigite, pyrite, and pyrrhotite) [81–86]. We found that addition of acetate, lactate, or glucose increased the potential for metabolic complexity with respect to C use and Fe(III) and sulfate reduction. Specific electron donors, such as glucose, can amplify the effects of initial community heterogeneity on the composition of the emergent membership when compared to simple electron donors (acetate and lactate) that have more constrained paths available (specific community members) in the community’s collective metabolism. These dynamic communities follow different geochemical pathways, depending on the specific electron donor provided, which has clear implications for ecosystem functions including C dynamics and greenhouse gas emissions, energy transfer processes linking terminal electron acceptor utilization and the biogeochemical cycles of major/minor elements, as well as contaminant fate and transport in both natural and engineered environments.

## Supporting Information

S1 Fig Picture of the microcosms at 124 d after inoculation. (PDF)

S2 Fig Picture of the repeat glucose microcosms at 50 d after inoculation and plots for each of the replicates of: glucose consumption and organic acid production; total and dissolved Fe(II) production and sulfate reduction; and pH. (PDF)

S3 Fig Results from XANES and EXAFS analysis of the bioreduced samples. (a) Normalized XANES. Top, spectra from several Fe(II) standards are overlaid on the starting ferrihydrite material (symbols). Below are the XANES spectra from systems incubated with different electron donors (black lines), showing the shift of the edge position to lower energy relative to the starting ferrihydrite material. The outlined small and large rectangles at the top-left delineate the regions of the derivate spectra shown in panels b and c. (b and c) Derivative of the XANES spectra. Top, starting ferrihydrite material (symbols) and standards. Below, comparisons between the starting material (symbols) and incubations with the different electron donors (black lines). In the incubation with acetate shown on panel C, arrows denote the shift from the starting material spectrum in the direction of the O-coordinated Fe(II) standards (7120–7125 eV). In the incubation with lactate, arrows denote the shift from the starting material spectrum in the direction of the S-coordinated Fe(II) standard (7120–7125 eV). (d) Linear combination fits of the derivative XANES spectra. Components and numerical results are discussed in the text. (e) Linear combination fits of the EXAFS spectra. Components and numerical results are discussed in the text.
(PDF)

S1 Table Summary of 454 sequence output per electron donor series. Amplicon libraries were constructed to target the V3-V4 region of the 16S rRNA-gene (*E*. *coli* positions 338–802).
(XLS)

S2 Table Summary of the total percentage of the microbial phyla by 454 based sequencing. Amplicon libraries were constructed to target the V3-V4 region of the 16S rRNA-gene (*E*. *coli* positions 338–802).
(XLS)
