# Supplementary figures and images for: Impact of Organic Carbon Electron Donors on Microbial Community Development under Iron- and Sulfate-Reducing Conditions
Source: PLoS One. 2016 Jan 22;11(1):e0146689. doi: 10.1371/journal.pone.0146689 (PMC4723079; doi:10.1371/journal.pone.0146689)

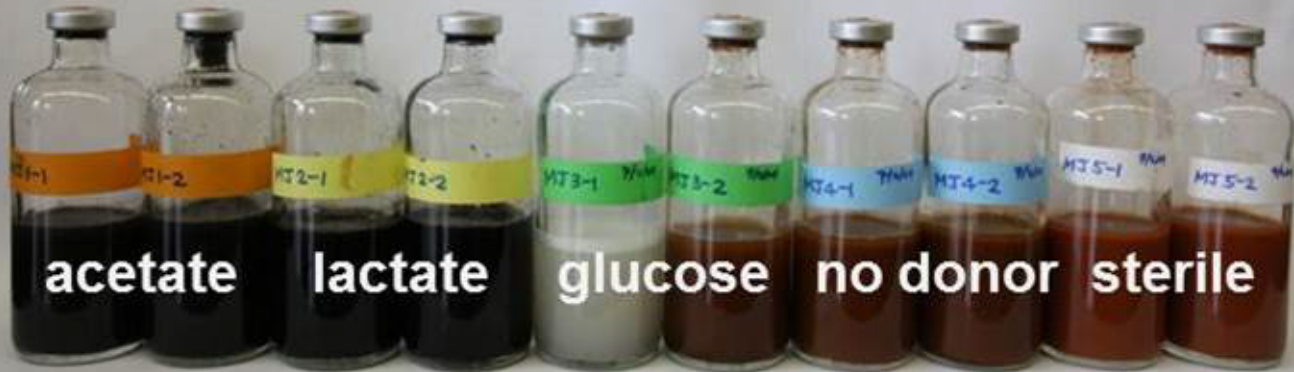

**acetate**

**lactate**

**glucose**

**no donor**

**sterile**

Supplement: S1 Fig — (PDF) [file pone.0146689.s001.pdf]

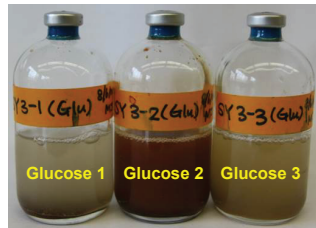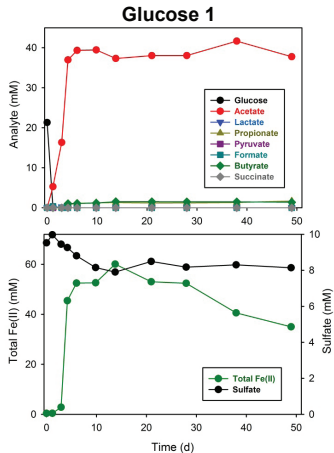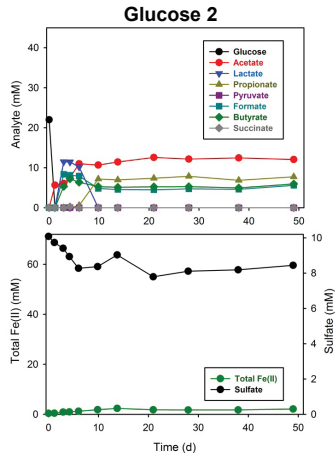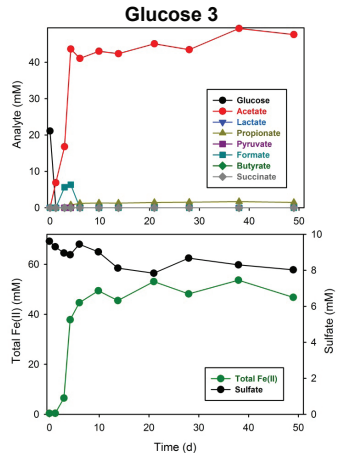

Supplement: S2 Fig — (PDF) [file pone.0146689.s002.pdf]

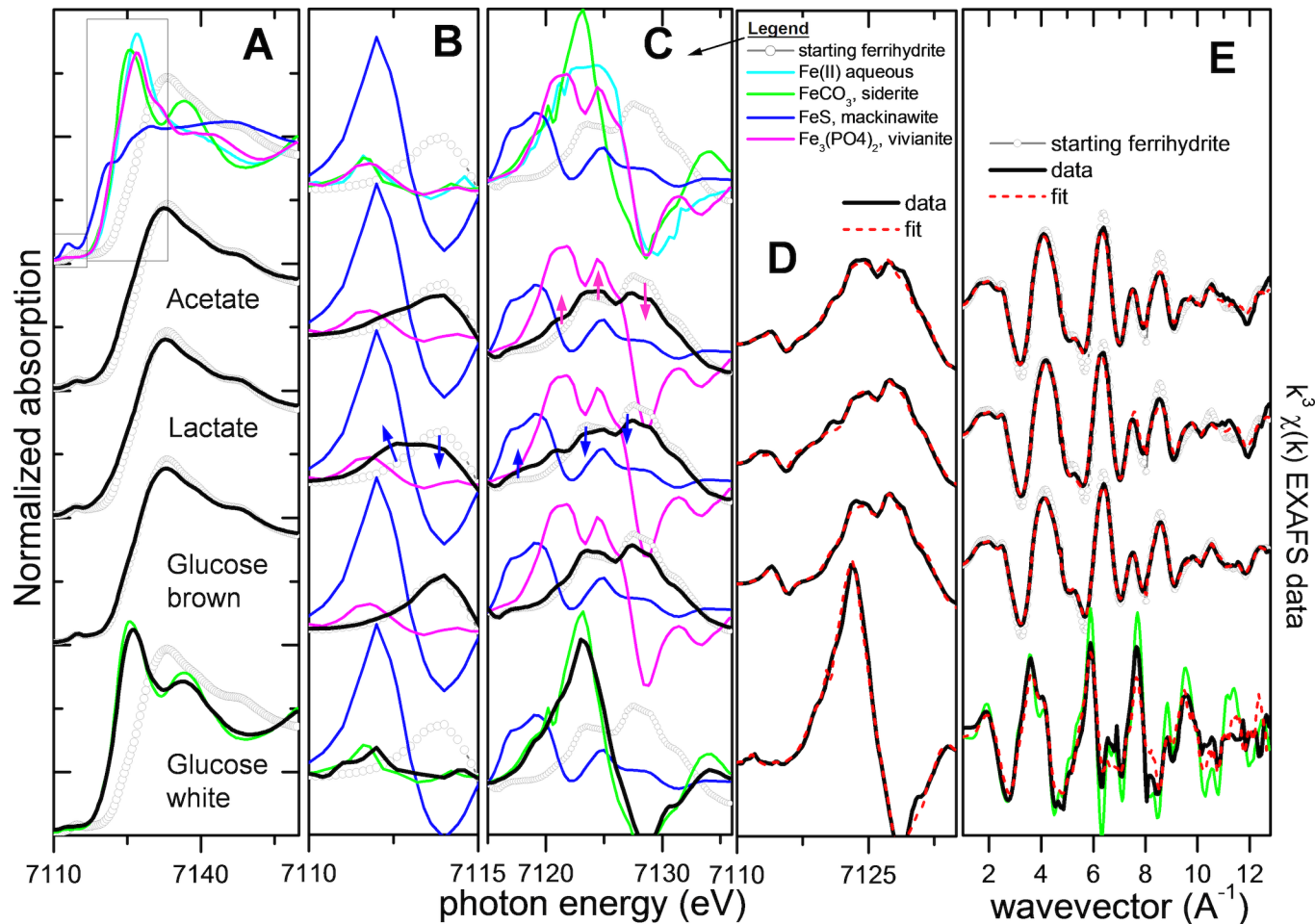

Supplement: S3 Fig — (a) Normalized XANES. Top, spectra from several Fe(II) standards are overlaid on the starting ferrihydrite material (symbols). Below are the XANES spectra from systems incubated with different electron donors (black lines), showing the shift of the edge position to lower energy relative to the starting ferrihydrite material. The outlined small and large rectangles at the top-left delineate the regions of the derivate spectra shown in panels b and c. (b and c) Derivative of the XANES spectra. Top, starting ferrihydrite material (symbols) and standards. Below, comparisons between the starting material (symbols) and incubations with the different electron donors (black lines). In the incubation with acetate shown on panel C, arrows denote the shift from the starting material spectrum in the direction of the O-coordinated Fe(II) standards (7120–7125 eV). In the incubation with lactate, arrows denote the shift from the starting material spectrum in the direction of the S-coordinated Fe(II) standard (7120–7125 eV). (d) Linear combination fits of the derivative XANES spectra. Components and numerical results are discussed in the text. (e) Linear combination fits of the EXAFS spectra. Components and numerical results are discussed in the text. (PDF) [file pone.0146689.s003.pdf]
